# Predictive and prognostic value of circulating blood lymphocyte subsets in metastatic breast cancer

**DOI:** 10.1002/cam4.1891

**Published:** 2019-01-10

**Authors:** Jian Yang, Junnan Xu, Ying E, Tao Sun

**Affiliations:** ^1^ Department of Medical Oncology, Cancer Hospital of China Medical University Liaoning Cancer Hospital & Institute Shenyang China

**Keywords:** CD3, CD4, lymphocytes, metastatic breast cancer, survival

## Abstract

The treatment of breast cancer (BC) has improved greatly in recent years, however, the limitations of current therapeutic modalities underscore the need to define new prognostic tools and develop highly targeted therapies. The aims of the present study were to explore the effects of circulating blood lymphocyte subsets on the survival of metastatic breast cancer (MBC) patients and to evaluate their predictive and prognostic value. The clinical data of 482 patients with MBC were retrospectively analyzed, and patients were grouped according to molecular types of BC. The distribution of peripheral blood lymphocyte subsets at the time of first metastasis was examined by flow cytometry, and the distribution of lymphocyte subsets in each group was categorized into ‘‘high or low’’ subgroups using the upper quartile point as the cutoff. The relationship between the distribution of lymphocyte subsets and progression‐free survival (PFS) as well as overall survival (OS) was evaluated in diverse molecular MBCs. In multivariate analysis, CD4^+^ was a negative independent predictor of PFS (hazard ratio [HR] = 0.538, 95% confidence interval [CI] = 0.313‐0.926, *P* = 0.025) and CD3^+^ was a poor independent prognostic factor for OS (HR = 0.437, 95% CI = 0.248‐0.772, *P* = 0.004) in the human epidermal growth factor receptor 2 (HER2)‐positive group. Neither the CD8^+^, CD19^+^, and CD56^+^ lymphocyte subsets nor the CD4^+^/CD8^+^ ratio in peripheral blood was significant predictive or prognostic factors. In conclusion, higher circulating levels of CD4^+^ and CD3^+^ at first diagnosis in HER2‐overexpressing MBC were significantly associated with worse survival outcomes. Low levels of plasma CD4^+^ and CD3^+^ were associated with increased anti‐HER2 benefit in HER2‐positive MBC. The present results indicate that these factors can be used as predictive and prognostic indicators of the outcome of patients with MBC.

## 1 INTRODUCTION

Breast cancer (BC) is one of the most common female malignant tumors worldwide and is the leading cause of cancer‐related death in women.^1^ Metastatic, BC is generally incurable. Recent advances in molecular biology and immunology indicate that differences and dynamic alterations in the tumor immuno‐microenvironment directly or indirectly affect the disease course and outcome.^2^ Although BC is not considered a highly immunogenic tumor, evidence supports the presence of a rich tumor immunoenvironment in certain BC subtypes, the prognosis of which is related to the activation of the immune reactive pathway.^3^ Some researchers have suggested that immune factors in the tumor microenvironment can be used as new independent prognostic indexes, and their prognostic value comparable to that of TNM staging.^4^. Currently, there is a growing interest in the BC microenvironment as a prognostic factor as well as a potential therapeutic target. Tumor infiltrating lymphocytes (TILs) have become a study hotspot. Although the role of TILs in the prognosis of cancer is controversial, a large body of literature supports that TILs are strongly associated with recurrence rates and survival in multiple solid tumors.^5^ Similar correlations are reported in BC, although the magnitude of the effect varies by disease subtype, and high levels of TILs are mostly evident in estrogen receptor (ER)‐negative and human epidermal growth factor receptor 2 (HER2)‐positive subtypes. In addition, studies show that TILs are associated with the beneficial effects of some treatments such as anti‐HER2‐targeted therapy and neoadjuvant chemotherapy.^3^, ^6^, ^7^, ^8^. The robust predictive ability of TILs for neoadjuvant chemotherapy is not restricted to ER‐negative cancers.^5^ An international cooperation is currently working on standardizing the evaluation of TILs to promote their implementation as biomarkers for BC. Ruffell et al^9^ reported that TILs in solid tumors are mainly composed of T lymphocytes and (CD3^+^) T lymphocytes predominantly consist of CD4^+^ T and CD8^+^ T cells. Therefore, changes in the number, distribution, or functional status of CD4^+^ T, CD8^+^ T, or total (CD3^+^) T cells may affect tumor outcomes. However, previous studies investigating TILs have mostly focused on early BC patients undergoing surgery or neoadjuvant chemotherapy and little information is available on TILs in advanced diseases. Furthermore, the detection of TILs is complex and cannot be dynamically monitored. In this context, the use of peripheral blood as the main source of immune cells in the tumor microenvironment^10^ has several advantages including simpler handling, easily accessible material, and the possibility of dynamic monitoring. In the present study, we analyzed lymphocyte parameters in circulating blood to provide basic data for further exploration of clinically useful tumor predictive and prognostic immune indicators.

## 2 OBJECTIVES AND METHODS

### 2.1 Patient data

This retrospective study was based on a consecutive series of 482 patients with metastatic breast cancer (MBC) who were diagnosed from March 2010 to March 2018 and hospitalized in our department. Case screening was performed according to the following criteria: Inclusion criteria: (a) The first metastasis was confirmed by pathology or imaging. (b) Clinicopathological information and survival data were complete. (c) Lymphocyte immune subpopulations data were complete within 3 months before and after the first metastasis. (d) Patients did not take drugs that may affect the body's immune function (such as immunopotentiators) or have immune‐related diseases (such as AIDS). Exclusion criteria: (a) Pathology, imaging, or other related examinations confirmed as non‐BC patients, or no metastases occurred by the deadline of the study. (b) Clinicopathological information or survival data were incomplete. (c) Lymphocyte immune subpopulations data were incomplete within 3 months before and after the first metastasis. (d) Patients took drugs that may affect the body's immune function (such as immunopotentiators) or had immune‐related diseases (such as AIDS). Eventually, 482 patients were enrolled and separated into four groups: luminal A group (n = 122), luminal B group (n = 220), HER2‐positive group (n = 75), and triple‐negative group (TN) group (n = 65). Baseline clinicopathological characteristics such as age, pathological type, tumor size, ER status, progesterone receptor (PR) status, HER2 status, Ki67 status, visceral invasion, multiple invasion, menstrual status, and treatment conditions are given in Table 1. Patients enrolled into the study were all female, with a median age of 55 years (23‐81 years). Follow‐up information was obtained by telephone. Until the last follow‐up period of March 10 in 2018, 187 patients died. The median follow‐up period was 42 months (range, 3‐96 months). The 2‐year progression‐free survival (PFS) rate and overall survival (OS) rate were 23.4% and 52.9%, respectively. All patients involved in the study were informed and consented to use their blood samples and related clinical data.

**Table 1:** Clinicopathological characteristics of 482 MBC patients with different molecular types

Characteristics	Luminal A N = 122	Luminal B N = 220	HER2 N = 75	TN N = 65
Age (y)				
＜50	24 (19.7%）	67 (30.5%）	25 (33.3%）	22 (33.8%）
≥50	98 (80.3%）	153 (69.5%）	50 (66.7%）	43 (66.2%）
Visceral invasion				
Yes	38 (31.1%）	94 (42.7%）	46 (61.3%）	18 (27.7%）
No	84 (68.9%）	126 (57.3%）	29 (38.7%）	47 (72.3%）
Multiple invasion				
Yes	28 (23.0%）	57 (25.9%）	20 (26.7%）	20 (30.8%）
No	94 (77.0%）	163 (74.1%）	55 (73.3%）	45 (69.2%）
Invasive ductal carcinoma				
Yes	87 (71.3%）	182 (82.7%）	62 (82.7%）	56 (86.2%）
No	35 (28.7%）	38 (17.3%）	13 (17.3%）	9 (13.8%）
ER				
Positive	120 (98.4%）	208 (94.5%）	0 (0%）	0 (0%）
Negative	2 (1.6%）	12 (5.5%）	75 (100%）	65 (100%）
PR				
Positive	102 (83.6%）	159 (72.3%）	0 (0%）	0 (0%）
Negative	20 (16.4%）	61 (27.7%）	75 (100%）	65 (100%）
HER2				
Positive	0 (0%）	89 (40.5%）	75 (100%）	0 (0%）
Negative	122 (100%）	131 (59.5%）	0 (0%）	65 (100%）
Ki67				
Positive	0 (0%）	201 (91.4%）	63 (84.0%）	47 (72.3%）
Negative	122 (100%）	19 (8.6%）	12 (16.0%）	18 (27.7%）
Tumor size (cm)				
T1 ＜ 2	62 (50.8%）	116 (52.7%）	29 (38.7%）	32 (49.2%）
T2 ≥ 2	60 (49.2%）	104 (47.3%）	46 (57.3%）	33 (50.8%）
Treatment lines				
First or second line	108 (88.5%）	194 (88.2%）	65 (86.7%）	56 (86.2%）
Multilines	14 (11.5%）	26 (11.8%）	10 (13.3%）	9 (13.8%）
Menstrual status				
Premenopause	102 (83.6%）	150 (68.2%）	50 (66.7%）	46 (70.8%）
Postmenopause	20 (16.4%）	70 (31.8%）	25 (33.3%）	19 (29.2%）

ER: estrogen receptor; PR: progesterone receptor; HER2: human epidermal growth factor receptor‐2; TN: triple‐negative group.

### 2.2 Prognosis assessment

For the survival analysis, the primary end point was PFS as defined by the time interval between the date of first cancer metastasis and the date of disease progression (PD) after the first metastasis or to death whenever death occurred before PD. OS was defined as the time period from the date of first cancer metastasis to the date of death or study deadline. PD was defined according to the revised evaluation criteria of the solid tumor in the 2009 (Response Evaluation Criteria in Solid Tumors, RECIST version 1.1).

### 2.3 Molecular assessment

Luminal A type: ER/PR positive and high PR expression; HER2 negative; Ki67 low expression. Luminal B type: (a) ER/PR positive; HER2 negative and Ki67 high expression or PR low expression. (b) ER/PR positive; HER2 positive; Ki67 in any status. HER2‐positive type: ER negative and PR negative; HER2 positive. TN type: ER negative; PR negative; HER2 negative.

### 2.4 Immunohistochemistry

Multiple biomarkers of breast tumors were detected by immunohistochemistry. Two pathologists carried out the readings independently and blinded from clinical outcome with the mean value of two evaluations used for the present analyses. The numbers of different lymphocyte subpopulations samples (CD3^+^, CD8^+^, CD4^+^, CD19^+^, CD56^+^, CD4^+^/CD8^+^ ratio, LymphEvents) in fresh peripheral blood were determined by antibody staining and flow cytometry. A series of monoclonal antibodies and flow cytometers were purchased from BD (BD Biosciences, Franklin Lake, NJ, USA) and operated according to reagent instructions.

### 2.5 Pathologic assessment

Basal phenotype positivity was defined as follows: ER/PR positive: tumor cell nuclear staining number ≥25% and/or 1+～3+; HER2 positive: IHC detection is 3+, and/or FISH detection represents gene amplification; Ki67 positive: tumor cell nuclear staining number ≥14% and/or 1+～3+.

### 2.6 Statistical analysis

All analyses were conducted by using SPSS 22.0 software (International Business Machines Corporation, Armonk, NY, USA) and Graphpad Prism 7.0 (GraphPad Software Corporation, San Diego, CA, USA). For each lymphocyte subpopulation: CD3^+^/LymphEvents, CD8^+^/LymphEvents, CD4^+^/LymphEvents, CD19^+^/LymphEvents, CD56^+^/LymphEvents, CD4^+^/CD8^+^ ratio, taking the upper quartile value of the proportion as the cutoff value and divide each subpopulation into two subgroups (record as high and low). The remaining covariates were grouped according to the classification criteria in Table 1. Kaplan‐Meier curves and log‐rank test were used for survival analysis. Multivariate analysis was performed by using the Cox proportional hazard regression model. Significant variables in univariate analysis (with *P* < 0.10) were retained for the multivariate procedure. Assessment of the associations in survival time between the received anti‐HER2 treatment and nonreceived anti‐HER2 treatment was performed by using Mann‐Whitney *U* test as appropriate. A two‐tailed sided *P*‐value <0.05 was considered significant.

## 3 RESULTS

### 3.1 Association with breast cancer subtypes

**Figure 1:**
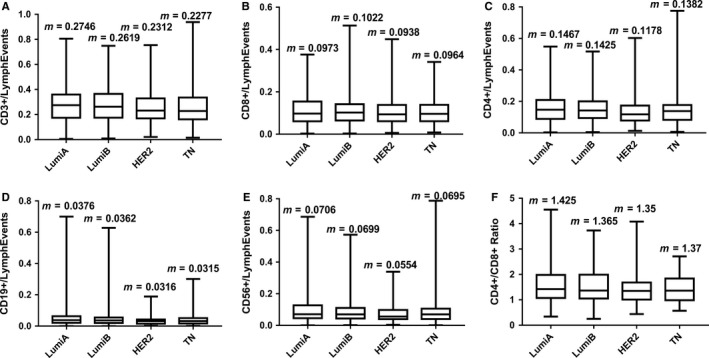
A‐E, Distribution of peripheral blood CD3^+^, CD8^+^, CD4^+^, CD19^+^, and CD56^+^ in different molecular types of MBC; F, Ratio of CD4^+^ to CD8^+^ in different molecular types of MBC. M is the median of the proportion of each lymphocyte subpopulation

Figure 1 shows box plots of circulating blood lymphocyte subsets in patients with different molecular types of MBC. The characteristics of the box plots for each lymphocyte subgroup and the corresponding median indicate that the lymphocyte populations are slightly higher in the luminal types than in the HER2 and TN types. Although the distribution of lymphocyte subsets is consistent with that reported previously,^11^, ^12^, ^13^ the differences in the distribution of lymphocytes between the molecular types were not statistically significant. Table 2 describes the survival time of BC patients with different molecular types. The results indicated that PFS and OS were slightly longer in the luminal groups than in the HER2‐positive and TN groups, suggesting that the variation in the survival time for each BC subtype followed a similar trend to lymphocyte distribution. However, the distribution of lymphocyte subpopulations in the HER2‐positive group was slightly lower than that in the TN group, whereas PFS and OS were better. This may be related to new drugs targeting the HER2 gene.

**Table 2:** Comparison of PFS and OS in different molecular types

Time/group	Luminal A	Luminal B	HER2	TN
PFS (m)	20	11	10	7
OS (m)	38	28	21	20

PFS: progression‐free survival; OS: overall survival; TN: triple‐negative group.

The PFS and OS of each group are shown by the median of survival time.

### 3.2 Association with patient outcome

Analysis of the relationship between the distribution of peripheral blood lymphocyte subsets and PFS and OS in 482 MBC patients showed that CD4^+^ (*P* = 0.026) and CD19^+^ (*P* = 0.014) were independent predictors of PFS in univariate analysis. CD4^+^ (*P* = 0.008) and CD19^+^ (*P* = 0.005) were also independent prognostic factors for OS. However, CD19^+^ and CD4^+^ were not significantly associated with patient outcomes in the multivariate analysis including age, pathological type, tumor size, ER status, PR status, HER2 status, Ki67 status, visceral invasion, multiple invasion, menstrual status, and treatment conditions (data not shown).

Because of variation in the prognosis between the different molecular types of BC, the 482 MBC patients were divided into four groups according to molecular criteria, to further analyze the potential relationship between lymphocyte subsets and survival.

### 3.3 Survival analysis in the HER2‐positive group

**Figure 2:**
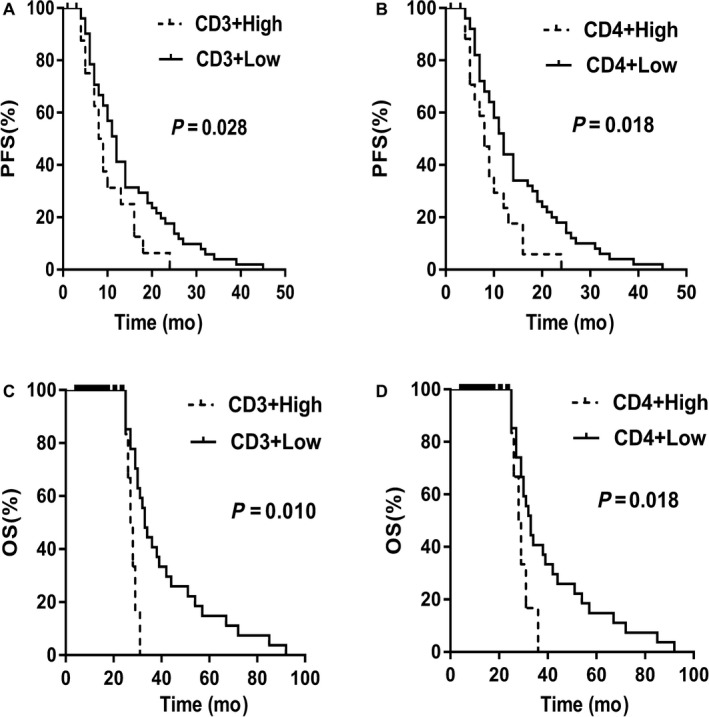
A, Relationship between the distribution of plasma CD3^+^ and PFS in the HER2‐positive group; B, Relationship between the distribution of plasma CD4^+^ and PFS in the HER2‐positive group; C, Relationship between the distribution of plasma CD3^+^ and OS in the HER2‐positive group; D, Relationship between the distribution of plasma CD4^+^ and OS in the HER2‐positive group

In patients with HER2‐overexpressing tumors, univariate analysis of PFS showed that CD3^+^ (*χ* ^2^ = 4.851, *P* = 0.028) and CD4^+^ (*χ* ^2^ = 5.570, *P* = 0.018) were independent negative predictors. The CD3^+Low^ and the CD4^+Low^ subgroups were significantly associated with longer survival. Similar results were obtained for OS, as CD3^+^ (*χ* ^2^ = 6.679, *P* = 0.010) and CD4^+^ (*χ* ^2^ = 5.551, *P* = 0.018) were independent poor prognostic factors for OS in the univariate analysis and the CD3^+Low^ and the CD4^+Low^ subgroups were significantly associated with better survival time. However, in the multivariate Cox model, only CD4^+^ was a negative predictor of PFS (hazard ratio [HR] = 0.538, 95% confidence interval [CI]:0.313‐0.926, *P* = 0.025) and CD3^+^ was a poor prognostic factor for OS (HR = 0.437, 95% CI: 0.248‐0.772, *P* = 0.004). The predictive function of CD3^+^ for PFS and the prognostic function of CD4^+^ for OS did not remain stable in the same multivariate analysis model. Taken together, these data indicated that patients with higher levels of circulating CD4^+^ and CD3^+^ at the first metastasis had a worse outcome than those with lower levels, and circulating CD4^+^ and CD3^+^ T lymphocytes in the blood of MBC patients can predict short‐term outcomes and determine the long‐term prognosis (Table 3 and Figure 2).

**Table 3:** Univariate and multivariate prognostic value of circulating lymphocytes in HER2‐positive disease for PFS and OS

Case	χ^2^	PFS			OS	
		Uni *P*	Multi *P*	χ2	Uni *P*	Multi *P*
Multiple metastases	0.834	0.361	—	4.550	0.033^a^	0.012^a^
Visceral metastases	0.817	0.366	—	4.194	0.041^a^	0.249
CD3^+^	4.851	0.028^a^	0.645	6.679	0.010^a^	0.004^a^
CD8^+^	2.367	0.124	0.658	2.275	0.131	0.856
CD4^+^	5.570	0.018^a^	0.025^a^	5.551	0.018^a^	0.711
CD16^+^CD56^+^	0.022	0.883	0.396	0.007	0.935	0.239
CD19^+^	0.147	0.701	0.535	0.141	0.707	0.283
4/8 ratio	0.252	0.615	0.421	0.000	0.984	0.747

PFS: progression‐free survival; OS: overall survival.

Variables with *P* < 0.1 in univariate analysis can be included in multivariate analysis. The table only shows the results of all immunological variables and other statistically significant variables.

a *P* < 0.05.

### 3.4 Survival analysis in luminal and TN groups

Univariate and multivariate analysis of PFS and OS in patients with luminal A, luminal B, and TN groups showed no statistically significant correlation between the distribution of peripheral blood lymphocyte subgroups and prognosis (*P* ≥ 0.05, data not shown). The CD3^+^, CD4^+^, CD8^+^, CD19^+^, and CD56^+^ lymphocyte subpopulations and the CD4^+^/CD8^+^ ratio in circulating blood had a lower predictive or prognostic value in luminal or TN types. The results obtained in luminal BC suggested that further research is warranted to examine the association between the immune system and the response to endocrine therapy.

### 3.5 Relationship between circulating CD4 ^+^ and CD3^+^ levels and anti‐HER2 benefit

HER2 overexpression is a molecular oncodriver in 20%‐25% of BCs and is associated with an aggressive clinical course and poor prognosis.^6^ However, recent advances in anti‐HER2‐targeted drugs have improved the clinical outcomes of patients with HER2‐positive disease. Despite this, the contribution of the host immune system to the efficacy of anti‐HER2 therapy remains poorly understood. We therefore evaluated the relationship between circulating CD4^+^ and CD3^+^ T lymphocytes and the benefits of anti‐HER2 therapies. The results of stratified analysis of 53 patients in the HER2‐positive group (n = 75) who received anti‐HER2 therapy are shown in Table 4, and the median was used as the cutoff value (defining upper and lower subgroups). The survival time of the lower subgroup was significantly longer than that of the upper subgroup, although the difference did not reach statistical significance. These results indicated that lower levels of circulating CD4^+^ and CD3^+^ were associated with increased benefit from anti‐HER2 therapy in HER2‐positive MBC.

**Table 4:** Relationship between plasma CD4 ^+^ and CD3^+^ distribution and anti‐HER2 benefit

	CD3^+^				CD4^+^			
	Upper	Lower	*Z*	*P*	Upper	Lower	*Z*	*P*
PFS(m)	9	12	−1.639	0.101	8	12	−1.693	0.091
OS(m)	20	23	−0.797	0.327	15	24.5	−1.950	0.051

PFS: progression‐free survival; OS: overall survival. PFS and OS are displayed with the median of survival time. Mann‐Whitney *U* test was conducted to detect differences in survival time between the two subgroups.

## 4 DISCUSSION

Breast cancer is a complex and heterogeneous disease. It is estimated that 1.38 million new BC cases are diagnosed every year worldwide, accounting for 23% of all new cancer cases. BC is the second most common female malignant tumor after lung cancer.^14^ The recent definition of gene expression characteristics has significantly improved the classification of BC; however, the prognosis of BC differs greatly according to subtype and the cause for this remains unclear. This has prompted investigators to seek more refined treatment strategies. Prognostic molecular markers of cancer have originally focused on carcinoma cells and improving prognosis by modulating the cell cycle and assessing proliferating genes. However, increasing evidence supports that factors in the tumor microenvironment have a major impact on treatment responses and clinical outcomes.^15^ This has shifted attention toward investigating nonneoplastic cells present in the tumor microenvironment such as stromal cells and immune cells, whose interactions with tumor cells affect the long‐term outcomes of patients.^16^ Clinical studies assessing whole‐tumor gene expression profiles have shown that high immune signals are associated with improved patient prognosis in a variety of cancers, including subtypes of BC, with the strongest correlation observed in the ER‐negative and HER2‐positive subtypes.^3^, ^7^, ^8^, ^9^ CD3 is a surface molecule present in all T cells, and it represents a mixture of T cells with activating and inhibiting characteristics. CD3^+^ T is mainly composed of CD4^+^ helper cells including T1 and T2, CD4^+^ regulatory T cells (Tregs), and CD8^+^ cytotoxic T lymphocytes (CTLs). Traditionally, CTLs have been recognized as host‐protective, and tumors with higher levels of infiltrating CTLs are associated with better patient survival. As central participants in the immune system, CD4^+^ T cells perform a critical role in recruiting, activating, and regulating many facets of the adaptive immune response.^17^ Despite the fact that all CD4^+^ T lymphocytes subsets do not contribute equally, they are all fundamentally important for tumor immunity.^16^ The distribution of each T lymphocyte subgroup varies greatly during different stages of tumorigenesis, suggesting that they may be determinants of tumor progression.^18^ The currently accepted concept is that Th1 cells mediate anti‐tumor immune response, whereas Th2 and Treg cells have immunoinhibitory properties.^19^, ^20^ In the early stages of tumorigenesis, anti‐tumor effects mediated by CTL and Th1 are dominant. As the tumor progresses, the number and proportion of tumor‐promoting T lymphocytes grow rapidly and become dominant. Among them, the most representative is Treg, which can increase its proportion to 20%‐40% of the total CD4^+^ T cells in intratumoral or peripheral blood.^21^, ^22^ Samanth et al^3^ confirmed that CD4 ^+^ T cells in the interstitial infiltration of BC are mainly Tregs and Th2.

In the present study, we showed that circulating T lymphocytes were significantly correlated with prognosis and therapeutic efficacy in the HER2 amplification subtype, and not in the luminal or TN subtype. This suggests that the prognostic value of circulating T lymphocytes for MBC is similar to that of TILs for early BC, as both are related to specific molecular subtypes. The present results indicated that CD4^+^ was associated with PFS, which is consistent with prior studies involving CD4^+^,^6^, ^22^, ^23^, ^24^ hence, this result is more credible. Lymphocyte subpopulations that maintain an immunoinhibitory setting in CD4^+^ (such as Tregs and Th2) should dominate based on the negative association with survival time. However, a high level of CD3^+^ was associated with worse OS. This result may be biased, because immunosuppressive subpopulations (such as Tregs and Th2) should predominate due to the negative correlation with OS, therefore, the CD4^+^ T lymphocytes which containing directly immunosuppressive subpopulations (such as Tregs and Th2) will be more statistically significant if CD3^+^ T lymphocytes were significant. However, our results failed to show the statistical significance of CD4^+^ for OS in the multivariate analysis. This may be due to the small sample size or bias caused by unpredictable factors. Multiple previous and present studies on BC support that the immune response is more obvious in the ER‐negative subtype^6^, ^7^, ^25^; therefore, we hypothesize that the immune system may be more influenced by hormone receptor status than HER2 protein overexpression, and it is very likely that the luminal type is not sensitive to immune signals. Regarding the fact that our results did not reach the significance reported previously in the TN group, one possible cause is that peripheral blood immune cells are affected by a range of immunological and nonimmunological parameters, and they are relatively too far from the tumor nests to produce an immune response as strong as that observed in the tumor area. Another possible cause is that HER2 signaling may be responsible for sustaining an immunosuppressive microenvironment and perhaps has a more profound effect on the immune system, which is not a feature of the TN type. Therefore, a separate analysis of each subtype is essential when studying the effects of immune‐related factors on the prognosis of BC, otherwise, obtained data may be biased and could represent a substitute of the disparities in prognosis between intrinsic subtypes.

Anti‐HER2‐targeted drugs are thought to act primarily via direct effects on tumor cells^7^. Despite significant research efforts, no clinically clear biomarkers exist currently to distinguish patients who derive benefit from or are resistant to anti‐HER2 regimens. Therefore, we further investigated the correlation between circulating CD4^+^ and CD3^+^ subpopulations and anti‐HER2 benefit and highlighted the role of T lymphocytes in the efficacy of anti‐HER2 treatment. Although we obtained statistically negative results, the survival time of the lower subgroup was significantly longer than that of the upper subgroup. Therefore, we have reason to believe that the lower distributions of circulating CD4^+^ and CD3^+^ T lymphocytes play a role in increasing the anti‐HER2 benefit in HER2‐positive diseases. The present data suggest that anti‐HER2‐targeted drugs work at least in part by modulating CD4^+^ T lymphocytes. This concept is supported by Perez^26^ who showed that advanced BC patients with HER2‐positive tumors exhibit a significantly increased frequency of circulating Tregs, and therapeutic intervention with trastuzumab leads to an overall reduction to normal levels in the frequency of Tregs. However, we failed to assess the frequency of Tregs in CD4^+^ T lymphocytes in our study, and further analysis of the composition of CD4^+^ T lymphocytes is warranted. Regarding our negative statistical results, they were likely due to the small sample size and selective bias, as only 26 patients (of 53 patients who received anti‐HER2 therapy) were treated with Herceptin as an advanced first‐line treatment. Others either selected Herceptin as a postoperative adjuvant therapy so that patients were already tolerant to Herceptin in the advanced stage, or they had received multiline treatments before Herceptin, besides there were four patients who received lapatinib treatment. These reasons may have affected the patients’ survival time, and our results were therefore insufficient to produce statistically significant differences.

The present study had several limitations. First, we were unable to evaluate the impact of the treatment received (such as chemotherapy) on the immune response prior to inclusion in the study. Further evaluation of the interaction between peripheral blood lymphocyte subsets and chemotherapy is needed. Second, the multivariate regression model in our study did not adjust for lifestyle factors (such as smoking or drinking), body mass index, or other parameters that potentially affect lymphocyte subsets. Thus, their inclusion in models may weaken the effect estimates. Third, the sample number in this study was insufficient and the results need to be confirmed in larger prospective cohorts. Fourth, our study did not detect TILs, and whether TILs are related to circulating T lymphocytes was not analyzed. To the best of our knowledge, there are few studies on the correlation of immune parameters in intratumoral, peritumoral, and peripheral blood, and the conclusions are inconsistent. Therefore, investigators continue searching for more refinement. Fifth, the superiority of any specific marker in a prognostic model mostly depends on applied cutoff points and heterogeneity, whereas the optimal cutoff point for different biomarkers depends on the clinical question. For example, different cutoff values may be required for distinct BC subtypes. We tried to select the optimal cutoff point based on the receiver operating characteristic curve, however, we observed a continuous relationship between lymphocyte distribution and prognosis, whereby we had to select the upper quartile point as the cutoff value. Further studies should analyze the cutoff values separately based on clinical practice to select the cutoff with the highest stratification value.

In conclusion, the present study provides new insight into T lymphocytes, suggesting their association with clinical outcomes and anti‐HER2 treatment benefits in HER2‐positive MBC. Despite the fact that multiple studies including our research suggest that changes in immune components are associated with prognosis, the clinical effects are limited. Further studies are required to determine how anti‐HER2 treatment alters the immune microenvironment and whether the addition of immune checkpoint inhibitors can further improve the clinical outcomes in HER2‐positive disease. Although the role of combining targeted anti‐HER2 therapies and checkpoint inhibitors is currently undefined, there is no doubt that immune checkpoint inhibitors may further activate the local immune system and this is an area of active research interest. The GeparSixto study^27^ shows a significant positive correlation between immune checkpoint markers (such as PD‐1, PD‐L1, CTLA‐4, and IDO1) and TILs; however, additional trials are necessary to determine whether immune checkpoint markers are also related to circulating T lymphocytes. Nevertheless, it is certain that quantification of immune parameters may help identify patients who may benefit mostly from immune‐related therapies. In addition, the immune response is very complex and a better understanding of the interaction between the immune response, intrinsic subtypes, and patient outcomes is necessary. Further analysis of the nature and functional roles of T lymphocytes and other immune subpopulations in tumor progression is warranted to obtain evidence for the clinical effectiveness of this biomarker.
